# The Impact of Psoriasis and Atopic Dermatitis on Quality of Life: A Literature Research on Biomarkers

**DOI:** 10.3390/life12122026

**Published:** 2022-12-05

**Authors:** Anna Balato, Alexander Zink, Graziella Babino, Dario Buononato, Charlotte Kiani, Kilian Eyerich, Stefanie Ziehfreund, Emanuele Scala

**Affiliations:** 1 Dermatology Unit, University of Campania “Luigi Vanvitelli”, 80131 Naples, Italy; 2 Department of Dermatology and Allergy, School of Medicine, Technical University of Munich, 80802 Munich, Germany; 3 Unit of Dermatology, Karolinska University Hospital, 17176 Stockholm, Sweden; 4 Department of Dermatology and Venereology, Medical Center—University of Freiburg, Faculty of Medicine, University of Freiburg, 79104 Freiburg, Germany; 5 Division of Dermatology and Venereology, Department of Medicine Solna, Center for Molecular Medicine, Karolinska Institutet, 17176 Stockholm, Sweden

**Keywords:** psoriasis, atopic dermatitis, quality of life, biomarkers

## Abstract

Psoriasis (PSO) and Atopic dermatitis (AD) are common inflammatory skin diseases that affect people of all ages globally. They negatively impact the quality of life (QoL) of patients in health-related aspects such as physical, psychological and mental functioning. Here, we conducted a review of studies relating to candidate biomarkers and indicators associated with QoL impairment in PSO and AD. Data research was performed using PUBMED and SCOPUS databases from inception to September 2022. Most of the included studies reported genomic or proteomic biomarkers associated with disease activity and QoL outcomes. Sociodemographic, clinical and therapeutic factors have also been implicated in deterioration of life quality in these patients. The inclusion of clinical characteristics, QoL impairment and co-diagnosis should be considered in drug development programs, since processing biomarkers based on an increased number of features in addition to drug class and disease will intensify the value of the biomarker itself, thereby maximizing the future clinical utility as a stratification tool.

## 1. Introduction

The entire dermatological field is experiencing substantial developments, and the general classification of chronic inflammatory skin diseases such as psoriasis (PSO) and atopic dermatitis (AD) is being rebuilt based on an improved understanding of their pathophysiology [1,2,3]. Nowadays, the need to identify clinically relevant biomarkers for disease progression, treatment response and quality-of-life (QoL) outcomes has become a scientific imperative. However, the identification, validation and transfer of a biomarker into clinical practice are complex and time-consuming, and despite many efforts regarding PSO and AD, no biomarker has yet been transferred into routine clinical practice [1,4]. Herein, we conducted a review to identify biomarkers and QoL-associated factors that might contribute to life quality impairment in either PSO or AD.

## 2. Materials and Methods

This study is a narrative review. PUBMED and SCOPUS databases were searched from inception to September 2022 with the search terms “psoriasis” and/or “atopic dermatitis” [all fields], the keywords “quality of life” or “biomarker”; and “AND” as operator. A total of 42 papers reporting the search terms in the title were selected. The studies were explored and documented with additional literature data. Overall, 139 articles were included in the body of this review. The selection of articles was performed independently by GB, DB, and SZ. All articles were reviewed by senior authors (AB, AZ, and ES) who also had the final decision in case of disagreement between the initial selectors.

## 3. PSO and AD: Common and Uncommon Denominators

### 3.1. Epidemiology and Clinical Characteristics

**Figure 1:**
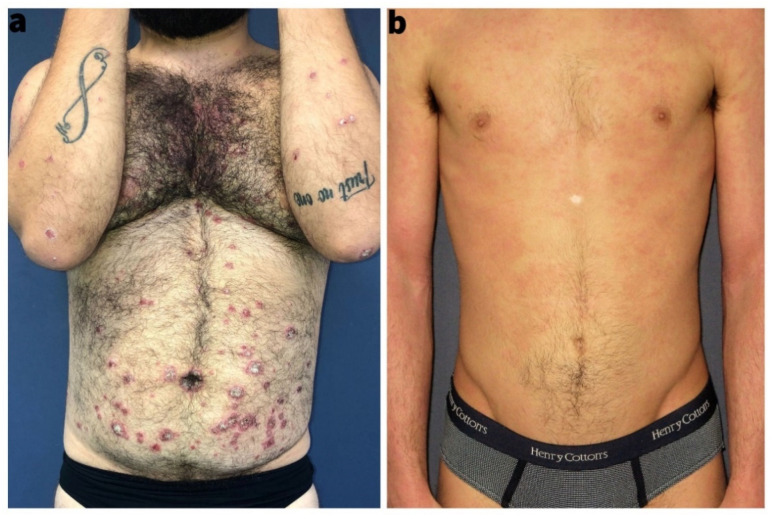
(**a**) Representative clinical image of a patient with plaque psoriasis; (**b**) representative clinical image of a patient with atopic dermatitis.

PSO and AD share clinical and epidemiological hallmarks. Both diseases are common with a prevalence ranging from 0.5% to 5% in adults all over the world; in children, the prevalence may be up to 25% in AD [5,6]. Both disorders are highly variable in terms of disease onset, severity, progression over time, and treatment response [1]. Clinically, PSO and AD present with red and scaly skin, but they differ in predilection sites: (i) PSO is common at extensor sites of extremities, umbilicus, and may present in an inverse phenotype axillary and inguinal region; (ii) AD typically presents at the flexure and in adults as a head-neck-shoulder type; both diseases frequently affect the scalp, hands, or feet, making a differential diagnosis in these areas challenging [3]. Morphologically, psoriatic lesions are sharply demarcated confluent papules, so-called plaques, whereas AD presents as a mixture of diffuse erythema, papules, vesicles, erosions, crusts, and fine lamellar scaling (Figure 1).

### 3.2. Genetics

Both PSO and AD are based at least partially on a genetic background. The putative chromosomes 1q21, 17q25 and 20p loci identified in AD are closely coincident with regions known to contain PSO susceptibility genes [7]. However, comparative studies revealed contrasting results, and most analyses are in favor of PSO and AD as opposing diseases [8]. The most frequent susceptibility gene locus of PSO is HLA-Cw6 (on PSORS1 6p21), whereas the null mutations of filaggrin (FLG) gene is the strongest genetic risk factor for AD [9,10,11]. HLA-Cw6 provided the initial evidence of a possibly (auto)immune component. With regard to the possible (auto)antigens, several hypotheses have been recently put forward. The keratinocyte-derived antimicrobial cathelicidin LL-37, for instance, was shown to form complexes with nucleic acids released by damaged cells and to cause the production of type I interferons by plasmacytoid and myeloid dendritic cells (DCs) [12,13]. Circulating cathelicidin (LL-37)-specific CD4 and CD8 T cells have consistently been identified in the blood of a majority of patients affected by moderate-to-severe plaque PSO [14]. Moreover, unbiased analysis of T cell receptors (TCRs) obtained from lesional infiltrating CD8 T cells revealed the presence of a CD8 T cell pool recognizing ADAMTSL5 (ADAMTS Like 5), a HLACw6 presented melanocyte antigen. Notably, ADAMTSL5 stimulation induced the PSO signature cytokine, IL-17A, supporting its role as psoriatic autoantigen [15]. Nowadays, several of the approximately 60 loci identified other than PSORS1 contains genes involved in the immune system at large and the Th17 pathway in particular: either upstream of the IL-17 expression, such as IL-23R and IL-12B, or downstream the IL-17 receptor, including STAT3 (signal transducer and activator of transcription 3) and Act1 (nuclear factor-κB activator 1) [16]. Back to AD, FLG is critically involved in the epidermal barrier, and its loss leads to increased permeability of the skin [10]. Besides FLG, more than 30 genetic loci have been identified to be associated with AD [17,18]. Additionally, variants in the IL-13 gene or the IL-6 receptor region and multiple rare protein-coding variants explain close to 30% of the total AD heritability [19].

## 4. Health-Related QoL and Psychological Aspects in PSO and AD Patients

The impact of these two skin diseases on health related QoL is considerable [20,21]. Skin disorders rank at the fourth position in the global burden of disease analysis regarding years lived with disability [21]. Psoriatic patients may feel stigmatized by their disease, and patients with AD are often anxious and have problems dealing with anger [20]. Both skin conditions can be triggered or worsened by mental disorders such as depression, anxiety, and sleep disturbance [22,23]. Moreover, people with PSO or AD are more likely to exhibit suicidal behaviors than healthy people [23,24,25]. However, the exact magnitude of the alleged association between these two skin conditions and psychiatric disease is still unknown. Growing evidence suggests that these mental disorders might be associated with some of biological aspects underlying PSO and AD pathogenesis, and in particular with systemic inflammation [26].

### Neuro-Immune Response in PSO and AD

The cumulative deterioration of QoL in PSO and AD patients has been linked to the crosstalk between the nervous system and the cutaneous immune cells. Immunologically, PSO is characterized by a Th17-induced activation of innate immunity including neutrophil migration into the skin and exaggerated metabolism and excessive keratinocyte proliferation, whereas AD is characterized by Th2 type immunity that leads to isotype switch towards IgE, recruitment of eosinophils and a decreased epidermal barrier and innate immunity [3,27]. Nevertheless, Asian, pediatric, and intrinsic types of AD involve Th17 as well [28].

Low-grade of systemic inflammation promotes and perpetuates metabolic changes of hormones implicated in cognition, emotion, and stress (e.g., serotonin, and melatonin) [22,29,30]. Indeed, several inflammatory mediators, cytokines and growth factors have been found in the plasma and central nervous system of individuals with depression and suicidal tendencies [31,32,33,34,35,36,37,38]. Such inflammatory factors include C-reactive protein (CRP), IL-6 and IL-17, tumor necrosis factor-α (TNF-α) and Toll-like receptors (TLRs) while among growth factors, there is vascular endothelial growth factor (VEGF). Interestingly, cytokine-targeted treatments were found to reduce symptoms of depression and anxiety in AD patients and in individuals with inflammatory conditions [39,40].

Systemic inflammation can disrupt circadian rhythm regulated by sleep hormone (melatonin), causing insomnia and sleep disorders [41]. In fact, this hormone is abolished or diminished in AD and PSO patients [42,43]. Melatonin supplementation has been demonstrated as an effective way also to improve AD severity in children [44]. Remarkably, it was able to reduce common pathological signs of PSO with the restoration of skin structural integrity, keratin content, and tight junction levels in vitro [45]. 

**Figure 2:**
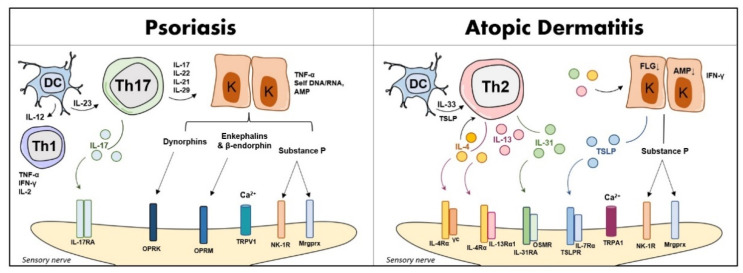
A simplified overview of non-histaminergic itch in PSO and AD. Abbreviations: AMP, antimicrobial peptides; DC, dendritic cell; FLG, filaggrin; IFN-γ, interferon-gamma; IL, interleukin; K, keratinocyte; OP, opioids; Mrgprx, Mas-related G-protein coupled receptor X; NK-1R, neurokinin 1 receptor; OPR, opioid receptor; OSMR, oncostatin M receptor; Th, T helper; TNF-α, tumor necrosis factor-alpha; TRPA1, transient receptor potential vanilloid 1; TRPV1, transient receptor potential ankyrin 1; TSLP, thymic stromal lymphopoietin.

Sleep quality disturbance has also been linked to pruritus, a symptom reported in both PSO and AD patients [46]. Indeed, itch determined by specific scores such as the ItchyQoL (a pruritus-specific QoL instrument) is comparable between PSO and AD patients [47]. In detail, keratinocytes and immune cells can release inflammatory mediators such as cytokines, histamine, and serotonin evoking itch in the skin [41,48,49]. Additionally, epidermal keratinocytes can release nerve growth factor (NGF) which causes sensitization of the skin to non-histaminergic itch [50]. However, this signaling is quite different in PSO and AD [11]: the sensory neuron substance P (SP), interleukin (IL)-2, calcitonin gene-related peptide (CGRP), opioid receptors (OPR)M, and OPRK are involved in PSO-related itch, while thymic stromal lymphopoietin (TSLP), CGRP, IL-4, IL-13, and IL-31 are associated with AD pruritus. Evidence on TSLP in PSO vulgaris has also been reported [51]. PSO itch is mainly induced by transient receptor potential vanilloid 1 (TRPV1) channel, but AD itch is mainly through transient receptor potential ankyrin 1 (TRPA1) [11] (Figure 2). 

## 5. The Need for Biomarkers to Predict QoL Impairment in PSO and AD

The need to identify clinically relevant biomarkers for disease progression, treatment response, as well as QoL outcomes, is nowadays of particular interest. In this scenario, the definition of biomarker given by regulatory organizations is particularly helpful, but not obviously universal, to pursue the research of new candidate markers.

The Food and Drug Administration (FDA) has proposed a rather broad definition: “A defined characteristic that is measured as an indicator of normal biological processes, pathogenic processes, or responses to an exposure or intervention, including therapeutic interventions”. The FDA also add the following comment: “Molecular histologic, radiographic, or physiological characteristics are types of biomarkers”; “a biomarker is not an assessment of how individual feels, functions or survives” [52]. The European Medicines Agency (EMA) has provided an even more restrictive definition of biomarker: “A biological molecule found in blood, other body fluids, or tissue that can be used to follow body processes and diseases in humans and animals” [53]. However, identifying and validating biomarkers for PSO and AD still requires understanding of many biological mechanisms and pathways underlying these two complex skin disorders. So far, candidate biomarkers and indicators have been associated with disease activity, treatment response and potentially to QoL outcomes in PSO and AD.

### 5.1. Candidate Biomarkers of Disease Activity, and Treatment Response in PSO and AD

Prognostic biomarkers of disease severity and progression have been investigated in PSO. A comprehensive catalogue of investigated biomarkers associated with PSO severity has been proposed, identifying LCE3D, IL23R, IL23A, NFKBIL1 loci and HLA-C*06:02 (genomic), IL-17A, IgG aHDL, GlycA, I-FABP, kallikrein 8 (proteomic) and tyramine (metabolomic) [54,55]. Moreover, six genomic biomarkers (HLA-C*06:02, HLA-B*27, HLA-B*38, HLA-B*08, and variation at the IL23R and IL13 loci), six proteomic biomarkers (IL-17A, CXCL10, Mac-2 binding protein, integrin b5, matrix metalloproteinase (MMP)-3 and macrophage-colony stimulating factor (M-CSF) and two metabolic biomarkers (tyramine and mucic acid) were selected as candidate biomarkers of PsA development in PSO [55,56]. However, no biomarkers were supported by sufficient evidence for clinical use without further validation. The risk of developing type 2 diabetes in PSO has been associated with eight variations in IL12B and IL23R loci [55]. A recent study revealed that metabolic aspects of PSO reinforce diabetes causing a greater cardiometabolic syndrome. Interestingly, biomarkers of insulin resistance, fasting plasma glucose (FPG) and fasting plasma insulin (FPI) were especially higher in diabetics with PSO compared with diabetics and controls [57]. Additionally, a group of biomarkers was described to evaluate the impairment of cardiovascular comorbidities in PSO: (i) Paraoxonase 1 (PON1) which exerts anti-atherogenic properties, was negatively correlated with liver enzymes activity predicting liver disorders in psoriatic patients [58]; (ii) Pentraxin 3 (PTX3), a marker related to heart failure and atherosclerosis, was elevated in the sera of psoriatic patients and negatively correlated with triglyceride, glucose and cholesterol levels [58]; (iii) N-terminal fragment of BNP precursor (NT-pro-BNP) which is strongly upregulated in cardiac failure, was higher in the sera of psoriatic patients than healthy controls emphasizing the correlation between PSO and cardiovascular disease [59]; and (iv) Chemokine ligand 20 (CCL20) was strongly associated with vascular endothelial inflammation, and it may serve as a potential biomarker of impaired vascular health in PSO [60].

Regarding systemic treatment response, candidate biomarkers of efficacy to TNF inhibitors (variation in CARD14, CDKAL1, IL1B, IL12B and IL17RA loci, and lipopolysaccharide-induced phosphorylation of NF-κB in type 2 dendritic cells) and ustekinumab (HLA-C*06:02 and variation in an IL1B locus) have also been identified [61]. Moreover, candidate biomarkers have been considered through several steps of the immune cellular crosstalk implicated in PSO pathogenesis, most notably antigen presentation, Th17 cell differentiation, positive regulation of NF-κB, and Th17 cell activation [61,62]. The candidate biomarkers identified require further evaluation to establish potential clinical utility according to most recent biological therapies. In fact, the majority of studies in PSO patients have investigated first-generation of biologics (TNF inhibitors and ustekinumab) as well as methotrexate, whereas new biomarker discovery and validation is a real unmet need urgent also for anti-IL 17 and anti-IL 23 therapies [61].

The AD course includes chronic relapse with skin inflammation and intense pruritus, which reduce patients’ QoL [63]. To date, 18 blood/serum biomarkers are known to be associated with disease activity of AD including % eosinophils in blood cell count, lactate dehydrogenase, total IgE, soluble IL-2 receptor, CCL17, CCL18, CCL22, CCL26, CCL27, IL-13, IL-22, IL-24, IL-25, IL-31, IL-33, TSLP, periostin and squamous cell carcinoma antigen-2 [64]. In particular, CCL18, TSLP and CCL26 were mainly associated with AD+ asthma phenotypes than AD alone in young children in a Danish study [65].

An exploratory clinical study has recently been conducted to determine which biomarkers are associated with good/poor clinical outcome of the anti-IL 4 receptor-α antibody dupilumab during the treatment of patients with moderate-to-severe AD. According to improvement of clinical symptoms, dupilumab has been significantly demonstrated to reduce the elevated expression of Th2 signatures such as IL-13, IL-31, Chemokine (C-C motif) ligand (CCL)17, CCL18, CCL22 and CCL26 in the blood and lesional skin of AD [66,67]. Even if these described biomarkers might be associated with treatment response to biological therapies, more studies are warranted to confirm this. In Table 1, we selected some of the described biomarkers associated with disease activity, treatment response and health related QoL impairment in PSO and AD.

### 5.2. Sociodemographic, Clinical and Therapeutic Factors Associated with QoL Impairment

It is now clear that the quantification of the impact of PSO and AD on patients’ QoL is important to obtain a complete picture. So far, a variety of QoL tools, including dermatology-specific and disease-specific QoL measures, have been developed and can be routinely implemented in the evaluation of patient’s life quality [78].

Established and validated instruments include the Short-Form 36-Item Health survey (SF-36), the PSO Disability Index (PDI) [79], the QoL Index for AD (QoLIAD) [80], the Children’s Dermatology Life Quality Index [81] and the most commonly used measure, the Dermatology Life Quality Index (DLQI) [82]. However, according to the WHO definition of health [83], it seems essential to additionally assess satisfaction-with-life (SWL) and happiness to obtain a more comprehensive evaluation of well-being and capture patients’ emotional burden [84,85]. However, hardly any studies are available in this for PSO and AD yet. 

There is evidence that dermatology-specific QoL but also SWL of PSO patients varies between countries, but also within different regions of a country [86,87,88,89]. Cultural differences, disparities in health care organization and access to treatment are subject of discussion for these regional differences [87] and should be considered in research and care. Moreover, lower education and income as predictors of impaired QoL [90,91,92] and SWL [93] may also be partly responsible for the differences.

More severe anxiety and depression symptoms, more intense itch and greater QoL impairment have been noted in psoriatic women [87,94,95,96]. Indeed, women with psoriasis have been shown to have worse disease related QoL than psoriatic men (evidence assessed by DLQI [89,91,97,98], Skindex [94] and PDI [90]), although higher disease severity has been found in men [94,95,97,98]. However, for SWL no gender differences were found by Kowalewska and colleagues [89]. Among AD patients, a study led by Holm found differences between men and women in the overall AD related QoL [99], other studies did not [100,101,102,103]. Interestingly, an association has been reported for QoL impairment in some CDLQI items, such as swimming/sports and teasing/bulling [101,102,103,104] and between DLQI and visible disease localizations in women with AD [105].

Controversial results are available regarding the association between age, disease duration, age of onset and QoL as well as SWL in psoriatic patients [89,90,91,92,97,98,106,107,108]. For example, some studies reported greater QoL impairment in younger individuals [90,106], while one study [89] reported higher SWL and better QoL in the youngest patients (18–30 years) and still others found no age differences [91,92,98]. AD can severely affect QoL of infants and children, especially social interaction, and tends to improve with age, although this can be moderated by sex, country of residence and disease severity and has been shown to differ in several QoL domains and items [20,99,100,101,109,110,111]. Raznatovic Durovic et al., reported that only the IDQOL item “child mood” positively correlated with age, whereas CDLQI total score and CDLQI item scores clothes/shoes, swimming/sports and sleep negatively correlated with age [111].

Based on DLQI but also various patient reported outcomes of interest, including SWLS and happiness, a significant association has been demonstrated between increasing AD severity, disease extent, eczema sites and impaired QoL, particularly social functioning and mental health [99,100,102,103,104,105,107,111,112,113]. Studies for PSO provide inconclusive results regarding the association between disease severity and QoL [90,96,98,106,108,114,115,116,117,118]. However, there is evidence that significant changes in PASI, affected body area and disease localization (e.g., facial or genital involvement) predict the DLQI and psychological and physical QoL [90,96,97,106,114,115,116]. Interestingly, PSO patients with complete clearance still have presented QoL impairment, which may be attributable to psychological conditions such as depression and anxiety [119]. Scientific evidence therefore indicates screening psoriatic patients for suicidal ideations and psychiatric disorders, with a focus on depression and anxiety, which are also well-known predictors of QoL impairment [106,115,120,121]. Joint and nail involvement, cutaneous comorbidities (e.g., vitiligo and AD) and metabolic syndrome symptoms have been shown to be associated with a reduction in QoL of patients as evidenced by DLQI, Skindex and PDI scores [88,122,123,124,125]. In contrast, there is evidence that higher BMI is associated with a better mental-health related QoL in psoriatic patients [96,126]. In AD, no association between QoL and atopic comorbidities has been reported [99,103,104].

Type of treatment has been shown to be a strong predictor of QoL among PSO and AD patients in clinical trials and real-life data. In detail, patients in receipt of biologic systemic therapy have been shown a better QoL compared to topical and conventional systemic treatment only [88,91,98,127,128,129,130,131]. In Table 2, the sociodemographic, clinical and therapeutic factors associated with QoL impairment in PSO and AD are summarized.

## 6. Conclusions and Future Directions

This review aimed to identify the current evidence on biomarkers associated with disease activity, therapy response and QoL outcomes in PSO and AD, serving as a key resource for the translational research community. However, the identification of clinically relevant biomarkers still needs to be elucidated in PSO and AD, therefore additional evidence is required also in light of different therapeutic targets that are now or will be available in the future. Furthermore, algorithms and risk prediction models including multiple biomarkers are likely to be required since a single biomarker is rarely likely to be a good predictor of disease activity and progression. The inclusion of clinical characteristics, QoL impairment and co-diagnosis should also be considered into drug development programs, since processing biomarkers on the base of ever more features apart from drug class and disease will intensify the value of the biomarker itself, thereby maximizing the future clinical utility as stratification tool.

With conventional methods, the identification of biomarkers often requires a prolonged analysis time and hospitals and laboratory setups with professionals. Digital tools enable new perspectives concerning disease progression, treatment response as well as QoL outcomes by real-time analysis with wearables. As still quite unknown mechanism there is the so-called Smart Skin technology. Smart Skin describes specific sensors worn directly on the skin, which can measure a wide range of parameters. From physical quantities (e.g., pressure, temperature, or humidity) to chemical parameters such as biomarkers in human fluids there are no limits [132,133]. Additionally, for biomarkers and factors associated with QoL impairment in PSO and AD this technique could be useful in future.

As already mentioned, itch is a common clinical symptom in both, PSO and AD, and has a big influence on the skin as well as on the psyche. Leading to worsened skin conditions, sleep disturbances and mental disorders, there is an unmet need for objectifying this symptom. Beneath specific scores like the ItchyQoL, Smart Skin sensors nowadays enable the possibility to quantify pruritus. By monitoring acoustic and mechanical signals with a sensor patch on the hand, the scratching behavior can be objectified. A clinical study with AD patients showed an accuracy of 99% versus visual observation; thus, with sensors like this, it will be possible to monitor disease severity or treatment response accurately and hence also jump to conclusions regarding the life quality of the patients [47,134].

Regarding the monitoring of biomarkers, the usage of whole blood and its derivatives (serum, plasma, etc.) is known as the gold standard. The method is well researched, but in the context of chronic diseases such as AD and PSO, it leads to repeated blood collections, which is uncomfortable for both the patient and the doctor, as it is time-consuming, painful and can be an entry point for pathogens [135]. Research on Smart Skin is increasingly focusing on the non-invasive and real-time analysis of medically relevant parameters in human biofluids such as saliva, urine or sweat [133]. Sweat could be a very promising biofluid for the tracking of inflammatory mediators and cytokines, as studies showed similar IL-6 and TNF-α concentrations between plasma and sweat [136,137]. In addition, the POWER study demonstrated a strong correlation between patients with major depressive disorder and the IL-6 and TNF-α sweat levels [138]. Additionally, CRP could already be quantified in human sweat [139]. Regarding those biomarkers as potential life quality impairment prediction factors in PSO and AD, this could be very relevant. A challenge in future will be the development of a mechanism to get an adequate amount of sweat, as perspiration may vary from individual to individual. Additionally, the concentration of the biomarkers may vary in sweat compared to blood, which demonstrates the need for very sensitive sensor systems and more research to know the differences.

## Figures and Tables

**Table 1:** Candidate biomarkers of disease activity and treatment response in PSO and AD, with relative features and therapeutics.

Biomarker	Main Points	Relevant Therapeutics
IL-6	Useful for assessing disease activity, developing of mental health in patients with PSO and for predicting responsiveness of joint symptoms to biologic treatments [37,68]	Tocilizumab
TNF-α	Correlates with disease activity, systemic treatment response in PSO and major depression [37,61,69]	Adalimumab, etanercept, infliximab
IL-17	Related to disease activity, systemic treatment response in PSO [70] and major depressive disorder [35]	Secukinumab, ixekizumab, brodalumab
IL-23	Correlates with depression, anxiety and disease activity in PsA [71]. It may serve for identifying joint activity or skin severity but not QoL or physical function [72]	Ustekinumab, guselkumab, risankizumab and tildrakizumab
HLA-Cw6	Associated with PSO severity and progression, as well as obesity and metabolic syndrome [54,73]	-
CCL20	Strongly associated with vascular endothelial inflammation, it reflects systemic inflammation and may serve as indicator of impaired vascular health in PSO [60]	-
PON1	Described as potential indicator of the liver disorders in PSO [58]	-
PTX3	Protective role regarding the development of cardiometabolic disorders, especially in overweight and obese patients with PSO [58]	-
CXCL10	Associated with the development of PsA among patients with PSO [56]	-
IL-4Rα	Correlates with disease activity and systemic treatment response in AD [66]	Dupilumab
IL-13	Related to disease activity and impairment of skin barrier in AD. It may activate itch signaling and scratching [64,74,75,76]	Tralokinumab
IL-31	Correlates with severity of allergic diseases [77] and good/poor clinical outcome of the anti-IL 4 receptor-α antibody dupilumab during the treatment of patients with moderate-to-severe AD [66]	Nemolizumab

Abbreviations: AD, atopic dermatitis; CXCL, C-X-C motif chemokine ligand; HLA, human leukocyte antigen; IL, interleukin; PSA, psoriatic arthritis; PSO, psoriasis; QoL, quality of life; R, receptor; TNF-α, tumor necrosis factor-alpha.

**Table 2:** Sociodemographic, clinical and therapeutic factors associated with QoL impairment in PSO and AD.

Factors	Psoriasis	Atopic Dermatitis
Sex	Women showed a higher QoL impairment compared to men [89,91,97,98]	NS difference in QoL impairment between genders [102,103,105]. However, female sex was associated with low QoL in a Danish study [99]
Age	Young patients showed higher QoL impairment in 2 studies [89,106]. NS differences in QoL impairment between age in 4 studies [91,92,98,108]	NS differences in QoL between young and adult patients by age [103]. Patients aged 16+ years showed a more impaired QoL than patients aged 4–15 years [99]
Place of residence	Urban areas showed more impaired QoL than rural areas [89]. Differences in QoL between 13 European countries: higher QoL in Spain, and lower QoL in Italy, especially in Southern Italy [87,88]	-
Educational level	A significant association was found between primary educational status and poor QoL [91]	-
Net salary	Lower income was associated with impaired QoL [92]	-
Disease duration/age at onset	Short disease duration had a higher impact on QoL in patients [98]. NS differences between impaired QoL and age at onset [76] or disease duration [92,108]	NS association between disease duration and QoL in patients with AD [113]
Disease Severity	Higher PASI score was associated with impaired QoL [92,98,108,115,116]	Higher SCORAD showed more impaired QoL [100,103,105,113]
Disease localization	Isolated involvement of scalp, trunk, intertriginous, palmoplantar and nail PSO was associated with a higher QoL impairment [88,106,125]	Isolated involvement of face, hand, genital and foot eczema was associated with low QoL [99,100]. Involvement of visible regions showed more impaired QoL than no involvement of visible regions in women [105]
Comorbidities	Patients with comorbidities such as hypertension, diabetes, lipid disorders, overweight/obesity, PSA, depression and anxiety had poorer QoL [98,106,124]	Adults with AD concomitant other atopic diseases including asthma, allergic rhinitis, allergic conjunctivitis experienced greater QoL impairment than the patients with AD alone [103]. NS differences in QoL impairment between patients with AD only and patients with comorbid atopic diseases [99,113]
Therapy	Topical therapy only was mostly associated with QoL impairment compared to topical therapy plus conventional systemic treatment [91]. Phototherapy and non-biological systemic therapy had a moderate effect on patients’ life, whereas biologics targeting TNF-α, IL-12/23 and IL-17 showed to improve patients’ QoL [88,98,131]	Topical corticosteroids only were mostly associated with QoL impairment compared to dupilumab therapy plus topical corticosteroids [128]

Note: Data obtained from studies using DLQI as outcome measure for assessing quality of life in patients with PSO or AD. Abbreviations: AD, atopic dermatitis; DLQI, dermatology life quality index; IL, interleukin; NS, non-significant; PASI, psoriasis area severity index; PSA, psoriatic arthritis; PSO, psoriasis; QoL, quality of life; SCORAD, scoring atopic dermatitis; TNF-α, tumor necrosis factor-alpha.

## Data Availability

Not applicable.
